# A Review of Public Health Emergency Preparedness and Response (PHEPR) Curricula in US CEPH-Accredited Schools and Programs of Public Health

**DOI:** 10.1017/dmp.2022.183

**Published:** 2022-09-16

**Authors:** Juliette Randazza, Jamie Vickery, Megan Archer, Lauren Dent, Claire Herrman, Amber S. Khan, Stephen C. Morris, Nicole A. Errett

**Affiliations:** 1 Department of Environmental and Occupational Health Sciences, University of Washington, Seattle, WA, USA; 2 Health Services Research, University of Rochester, Rochester, NY, USA; 3 Department of Emergency Medicine, University of Washington, Seattle, WA, USA

**Keywords:** disaster preparedness and response, public health education and training, health emergency

## Abstract

**Objective:**

To assess the current state of graduate-level disaster-related curricula (i.e., Masters and Doctoral programs, degree concentrations, and graduate certificates) offered by the Council on Education for Public Health (CEPH)-accredited public health schools and programs in the US.

**Methods:**

This research reviewed, evaluated, and summarized the content of websites of all US-based CEPH-accredited schools and programs to identify disaster-related degree programs, degree concentrations and graduate certificates from April – June 2021.

**Results:**

Of 191 schools and programs reviewed, 29 (15%) offered disaster-related curricula, totaling 44 degrees and programs. Programs included Masters-level degrees and Masters/Doctoral degree concentrations, with the majority taking the form of graduate certificates (64%). Schools that offered disaster-related curricula were clustered in eastern and Gulf states.

**Conclusion:**

Most US CEPH-accredited schools and programs do not offer graduate-level disaster-focused curricula. Of the programs offered, far fewer opportunities exist for in-depth graduate-degree level training compared to certificate-level training. Additionally, programs are constrained to certain areas of the country. Our findings suggest a need for disaster and public health emergency-related curricula development within schools and programs of public health to meet the growing needs of communities affected by disasters and emergencies.

## Introduction

In appreciation of the centrality of public health emergency preparedness and response (PHEPR) to public health practice, ‘Emergency preparedness and response’ is identified as 1 of 7 foundational capabilities of the Foundational Public Health Service framework, which outline government public health responsibilities, fundamental capabilities and services.^1^ Over the past few years, there has also been an increasing recognition of the importance of evidence-informed PHEPR practices, and the need for science and research to be fundamental components of PHEPR practice.^2,3^ Echoing these calls, the Centers for Disease Control and Prevention sponsored the National Academies of Sciences, Engineering and Medicine (NASEM) consensus study report, ‘Evidence-Based Practice for Public Health Emergency Preparedness and Response,’ and outlined a National PHEPR Science framework to improve the identification and implementation of evidence-based PHEPR practices.^4^ The framework calls for PHEPR to be recognized as a unique academic discipline within public health, and a robust commitment to PHEPR training for both practitioners and researchers.

In 2010, the Association of Schools and Programs of Public Health (ASPPH) created a public health preparedness & response core competency model for workforce competencies that ‘demonstrate the abilities that not only a public health professional involved in preparedness work must display, but which are recommended for all members of the public health workforce to enhance their readiness to respond to public health emergencies and disasters.’^5^ This includes ‘foundational public health,’ ‘generic health security or emergencies,’ ‘position-specific or professional’ competencies, as well as specific competencies under 4 domains: (1) model leadership, (2) communicate and manage information, (3) plan for and improve practice, and (4) protect worker health and safety. Although previous work has assessed disaster medicine knowledge, confidence, and attitude among students in health-related fields,^6^ as well as efforts towards creating educational frameworks for integrating disaster medicine competencies into health professions,^7^ little work has been done to understand if schools or programs of public health have instituted disaster-specific public health competencies and curricula to meet these demands in public health education.

In response, an assessment of Council on Education for Public Health (CEPH)-accredited schools and programs of public health gauged commitment to PHEPR training and education within the US, as well as the trajectory of PHEPR science as a unique academic discipline within public health. This type of assessment is necessary to understand the breadth and depth of current PHEPR training and to identify gaps in educational programming and competencies. Outcomes of this work can inform directions and opportunities for PHEPR capacity-building within CEPH-accredited schools and programs, which are essential toward the development and enhancement of PHEPR workforce capability and capacity. Findings of this assessment may also be applicable to non-CEPH accredited schools and programs.

## Methods

### Sampling & data collection

A total of 3 teams of 2 reviewers assessed the websites of 191 US based CEPH-accredited schools and programs to identify graduate-level programs with degree, degree concentration, and certificate offerings *explicitly* centered on disasters and health, and public health emergencies. (Nine international schools accredited by CEPH at the onset of review (April 2021) were omitted from the analysis. ASPPH competencies released for U.S. contexts was the basis of the analysis.) CEPH is the accrediting body for schools and programs of public health, as recognized by the US Department of Education, and is a member of the Association of Specialized and Professional Accreditors. For a program to receive CEPH-accreditation, they must undergo rigorous internal and external evaluation, including intensive peer review to determine if their policies and practices adhere to the standards of excellence in public health education and training.^8^ The data collection protocol and tool used to capture disaster-related graduate programming was structured so that each ‘entry’ represented a program (e.g., masters or PhD), concentration or certificate; on occasion, institutions had more than 1 unique entry depending on the number of programs, concentrations, and/ or certificates within a given CEPH-accredited school or program. To ensure reliability of the data collection protocol and tool (see Supplemental Material), the 6 reviewers first conducted a pilot of the tool by assessing the websites of the same 5 CEPH-accredited programs and 5 CEPH-accredited schools independently. Reviewers convened and discussed any discrepancies so they could refine the data collection process and update the tool to ensure clarity and ease of use.

In addition to carefully reading through each of the 191 US CEPH-accredited school and program websites, the reviewers used the following search terms in the website’s search bar to ensure they captured offerings pertaining to our research objective: ‘disaster,’ ‘public health preparedness,’ ‘emergency management,’ ‘health security,’ ‘humanitarian,’ ‘hazard mitigation,’ and ‘response.’ Each individual reviewer first assessed each identified program to determine if it met the following inclusion/ exclusion criteria:

### Inclusion criteria:

Program must be part of a CEPH-accredited school or program located in the US, must explicitly address disasters and public health emergencies through defined curricula, and must be actively accepting applications.

### Exclusion criteria:

Program is *outside* of public health schools or programs, or program materials (e.g., website description, recruitment material etc.) only reference disasters as part of a public/environmental health focus.

Team members abstracted specific data elements about included programs and entered this information into a REDCap web application form (Vanderbilt University, Nashville, Tennessee, USA). REDCap (Research Electronic Data Capture) is a secure, web-based application designed to support data capture for research studies, providing: 1) an intuitive interface for validated data entry; 2) audit trails for tracking data manipulation and export procedures; 3) automated export procedures for seamless data downloads to common statistical packages; and 4) procedures for importing data from external sources^9^. Prior to the search, the team examined a selection of graduate-level disaster and health programs to identify data elements of interest based on the information available on the program web page. Each team of 2 then met to compare the programming (degree concentrations, degree programs, and certificates) they included and the data they abstracted. Where there were discrepancies, they re-reviewed the school/ program website and attempted to achieve a consensus determination. For instances where the team could not reach consensus (e.g., because the program description was vague), study team members had to decide. Once each of the 3 teams adjudicated their unique set of entries in REDCap form, these were combined into a single spreadsheet for a second round of adjudication by 2 team members (JR and JV) who re-assessed the list of entries twice to ensure that they aligned with study inclusion and exclusion criteria.

Data gathered from each website include, when applicable, program type, program competencies and descriptions, options for online completion, thesis, practicum, or capstone requirements, and departments which offered the degree or program. Using this data, the team categorized the types of programming by institution, and the locations of schools were mapped to determine the geographical distribution of programs offered. Our assessment did not involve data collected from or about human subjects hence we did not require human subjects’ approval.

## Results

**Figure 1.**
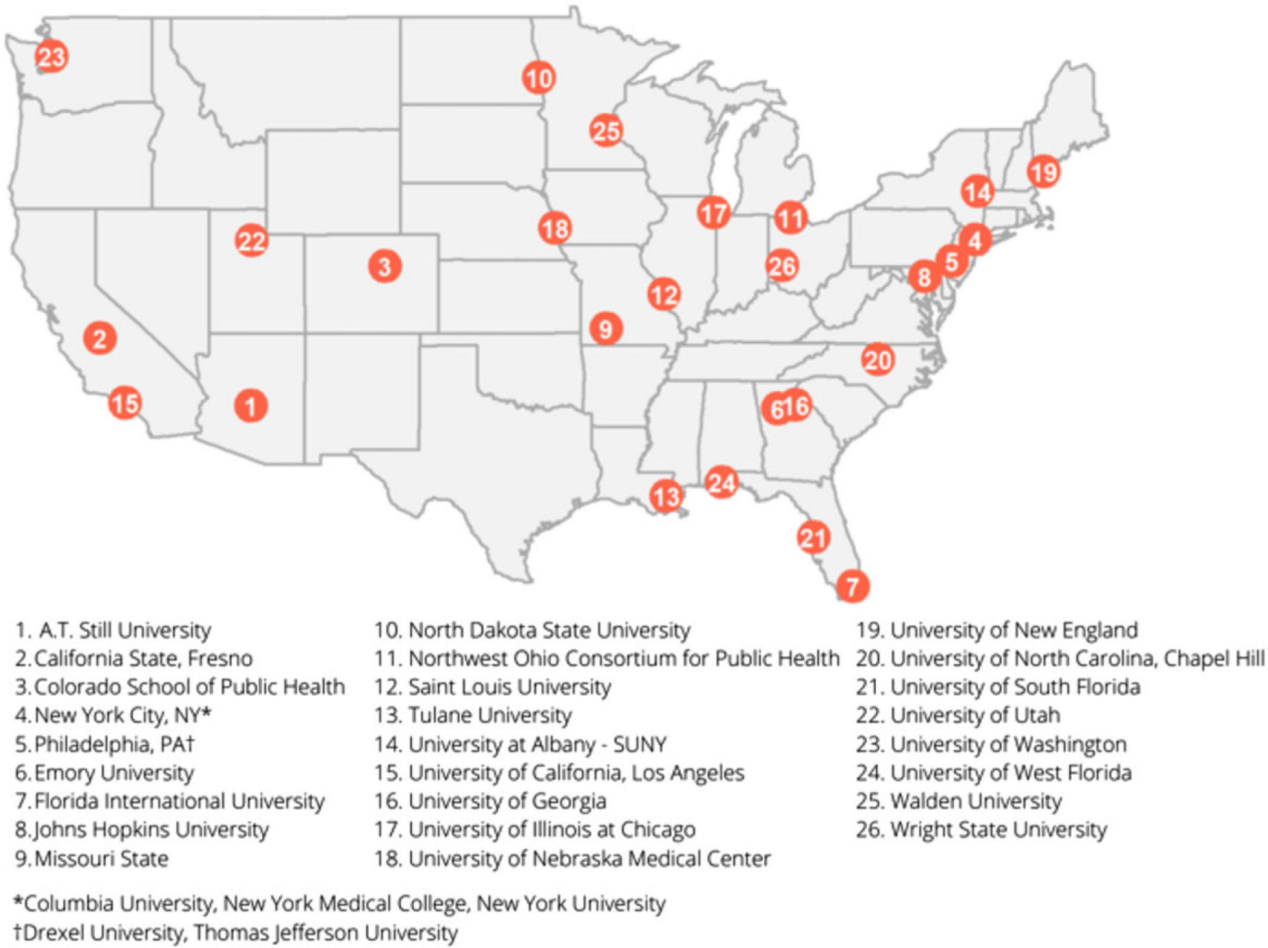
Map of Institutions with disaster-related public health programming.

A total of 44 concentrations, degree programs, and certificates (each representing a program entry) from 29 institutions were identified. The map in Figure 1 highlights institutions with PhD, MPH, MS, MA, graduate degree concentrations, and/ or certificate programs focused on PHEPR. While CEPH-accredited schools and programs with PHEPR-related training span the contiguous US, notable geographic gaps exist in terms of PHEPR offerings (e.g., Texas and western states).

Table 1 provides a visual of disaster-related public health training by degree/ program type. Only 7 institutions had multiple programs: Johns Hopkins University (n = 7), Northwest Ohio Consortium for Public Health/ University of Toledo (n = 2), Saint Louis University (n = 2), Tulane University (n = 3), University of Georgia (n = 2), University of Nebraska Medical Center (n = 3), and the University of South Florida (n = 3).

The 3 ‘other’ responses included a graduate minor, a Master of Applied Science in Humanitarian Health, and an MS /MPH in Disaster Medicine & Management/ Master of Public Health. The number of graduate certificate programs (n = 28) that exist was higher than other disaster training-related programming (n = 16) combined. Out of the 44 entries, less than 50% (n = 19) had competencies available on their website, such as how to assess an emergency to determine public health assistance needs and identification of threats to public health and public health considerations during and following disaster. Entries with publicly available competencies include: 4 programs with masters-level concentrations, 1 program with a PhD-level concentration, 11 certificate programs, 1 MPH degree, 1 MS degree, and 1 of the ‘other’ entries.

## Discussion

Although PHEPR training exists within certain CEPH-accredited schools and programs of public health, these findings suggest that additional programming and capacity-building are needed to prepare the next generation of the public health workforce to meet the increasing demand for evidence-informed PHEPR. Given that the limited number of PHEPR training opportunities within CEPH-accredited schools and programs is centered around graduate certificate offerings, as opposed to masters- and PhD-level degree programs, this signals a need for additional and meaningful commitment to the development of PHEPR training and education. This should entail a focus on curricula informed by current and future public health practice needs as well as research-related competencies for building and evaluating PHEPR evidence.

These findings indicate both programmatic and geographic disparities in access to PHEPR education. Schools and programs offering PHEPR-focused training are concentrated in the Gulf coast region and eastern US states. Reasons for these disparities could be a result of the number of higher education institutions in these areas, more local or regional exposure to emergencies and disasters, or differences in available capacity, and interest to support such programming across institutions. Notably, there are no schools or programs offering such training in Texas, even though Texas is 1 of the most disaster-affected states in the US. This poses a concern in terms of regional disparities in the ability to effectively build out local and regional PHEPR workforces to respond to disasters and public health emergencies. Schools and programs of public health in areas without substantial PHEPR curricula should prioritize its development, ideally in collaboration with public health agencies to contribute to PHEPR capacity development in the region.

The COVID-19 pandemic, and the increasing severity and frequency of disasters has highlighted the critical roles and responsibilities of the PHEPR workforce and underscores the need for such robust education and training. However, the most recent PHEPR competencies developed by the ASPPH are over a decade old.^5^ In response to recent calls for PHEPR as a unique academic discipline, updated PHEPR workforce competencies are necessary. Based on the need and opportunity to develop curricula reflected in these findings, particularly at the masters and doctoral levels, the development of such competencies should be a priority. Sustained federal resources are necessary to ensure these are informed by a broad community of practice, including both researchers and practitioners, and evaluated to ensure they reflect the dynamic PHEPR needs of the public health workforce in the coming decades.

As articulated in the 2020 PHEPR Consensus Study Report, ‘the gap between PHEPR research and practice can be narrowed by training researchers in translation and implementation science and supporting workforce development programs that strengthen the implementation capacity of public health agencies.’ Graduates with such competencies will be better equipped to address the research and practice needs for communities at risk of or affected by disaster and will ultimately contribute to our capacity for effective public health disaster response. In response to calls for increasing evidence about the health impacts of disasters and the effectiveness of public health response and recovery strategies, the National Institutes of Health developed the Disaster Research Response program (DR2) in 2014.^10^ Part of the focus of the DR2 program is to encourage training and education for students and scholars who may conduct research in public health emergency settings or disaster-affected areas. While the DR2 program has made enormous strides in the development of research infrastructure, including its RAPIDD protocol, community of practice and training workshops, future efforts focused on the development of curricula and training (e.g., through R25 or T32 funding mechanisms focused on training) may springboard additional investments.

### Limitations

This cross-sectional assessment only reflects information publicly available from online websites on *CEPH-accredited* schools and programs of public health in spring 2021. As such, the degrees, concentrations, and certificates captured may not represent the full range of existing disaster-related public health programs (e.g., at schools and programs of public health that have not undergone the CEPH-accreditation process, and disaster programs in other health sciences schools or related disciplines). However, a 2018 study found that from 1992 – 2016, CEPH-accredited institutions conferred more than 80% of all graduate-level public health degrees.^11^ The decision to use CEPH-accreditation as an inclusion criteria captures the schools and programs that confer the majority of graduate-level public health degrees in the US. In addition, while the titles and/ or descriptions of the programs, concentrations, and certificates focused on PHEPR, future qualitative work should review and summarize their competencies to identify alignment with the 2010 ASPPH PHEPR competencies to assess uptake, alignment, and gaps. Moreover, PHEPR curricula from non-CEPH accredited schools and programs may also provide opportunities for PHEPR competency development, suggesting the need for additional research.

## Conclusion

Few (29 out of 191) CEPH-accredited schools and programs of public health offer disaster and public health emergency-specific curricula. Given the increasing need for a competent, well-trained PHEPR workforce, additional programming at the graduate level (e.g., MPH, PhD) is needed. Current and future public health workforce needs should drive disaster-related public health training and education, as well as an infrastructure to collaboratively generate and implement evidence-based PHEPR practice.

## Supplementary Material

1

## Figures and Tables

**Table 1.** Disaster-related public health programming by institution

Institution	Master of Arts (MA)	Master of Science (MS)	Master of Public Health (MPH)	Masters-level concentration (incl. MPH)	Doctoral-level concentration	Certificate	Other	TOTAL (n)
**TOTAL (n)**	**1**	**2**	**2**	**7**	**1**	**28**	**3**	**44**
AT Still University						✓		1
California State, Fresno						✓		1
Colorado School of Public Health						✓		1
Columbia University						✓		1
Drexel University							✓	1
Emory University						✓		1
Florida International University	✓							1
Johns Hopkins University				✓✓✓	✓	✓✓	✓	7
Missouri State						✓		1
New York Medical College						✓		1
New York University						✓		1
North Dakota State University			✓					1
Northwest Ohio Consortium for Public Health						✓✓		2
Saint Louis University				✓		✓		2
Thomas Jefferson University							✓	1
Tulane University			✓			✓✓		3
University of Albany - SUNY						✓		1
University of California, Los Angeles						✓		1
University of Georgia				✓		✓		2
University of Illinois at Chicago						✓		1
University of Nebraska Medical Center		✓		✓		✓		2
University of New England						✓		1
University of North Carolina, Chapel Hill						✓		1
University of South Florida				✓		✓✓		3
University of Utah						✓		1
University of Washington						✓		1
University of West Florida						✓		1
Walden University		✓						1
Wright State University						✓		1
