# Supplementary material for: A Review of Public Health Emergency Preparedness and Response (PHEPR) Curricula in US CEPH-Accredited Schools and Programs of Public Health
Source: Disaster Med Public Health Prep. Author manuscript; Available in PMC 2023 Mar 16. (PMC9905216; doi:10.1017/dmp.2022.183)
Supplement: 1 [file NIHMS1825688-supplement-1.pdf]

Record ID	______________________________
Reviewer pair	______________________________
Name of school (ensure that this name is consistent with the name in the google sheet, if multiple schools make note at the end of the form):	______________________________
Graduate degree level:	◯ Master's degree<br>◯ Doctoral degree<br>◯ Certificate program<br>◯ Other
Program type:	◯ Master of Public Health (MPH)<br>◯ Master of Science in Public Health (MSPH)<br>◯ Master of Science (MS)<br>◯ Master of Health Administration (MHA)<br>◯ Master of Health Science (MHS)<br>◯ Doctor of Philosophy (PhD)<br>◯ Doctor of Public Health (DrPH)<br>◯ Certificate Program<br>◯ Other<br>◯ Don't know<br>◯ Concentration<br>◯ Master of Arts (MA)
If other, record program type:	______________________________
If concentration, check all programs that the concentration can apply to:	☐ Master of Public Health (MPH)<br>☐ Master of Science in Public Health (MSPH)<br>☐ Master of Science (MS)<br>☐ Master of Health Administration (MHA)<br>☐ Master of Health Science (MHS)<br>☐ Doctor of Philosophy (PhD)<br>☐ Doctor of Public Health (DrPH)<br>☐ Other<br>☐ Don't know
If other, please specify:	______________________________
Program name (ex. MPH in Disaster Management)	______________________________
If this is a certificate program, is this program offered only to degree students?	◯ Yes<br>◯ No<br>◯ Don't know
Is this certificate designed as a continuing education program to be taken by non-matriculated students?	◯ Yes<br>◯ No<br>◯ Don't know

Is this certificate a summer course designed for non-students?	◯ Yes<br>◯ No<br>◯ Don't know
Does the program have online components?	◯ Yes<br>◯ No<br>◯ Don't know
To what extent is the program online? Check all that apply:	☐ Online only<br>☐ Partially<br>☐ Don't know
Please describe what portions of the program are online (ex. courses, in residence discussion/components):	____
Does the program site include a list of competencies or educational objectives?	◯ Yes<br>◯ No<br>◯ Don't know
Please list these competencies:	____
Does the program include a description that is not a list of competencies?	◯ Yes<br>◯ No<br>◯ Don't know
Please copy and paste the program description into the text box:	____
Does the site list coursework required for the program?	◯ Yes<br>◯ No<br>◯ Don't know
Please include the link to the site where coursework is listed:	___________________________________
Include the names of the courses (with the number of credits if available):	____
Does the site include links to the course descriptions/syllabi?	◯ Yes<br>◯ No<br>◯ Don't know
Please provide links to the course descriptions/syllabi:	____
Is the program offered out of a specific department?	◯ Yes<br>◯ No<br>◯ Don't know
If yes, please list the department(s):	___________________________________

Is the program associated with a Center with research related to disasters, public health preparedness, health security, humanitarian studies, etc.?	◯ Yes<br>◯ No<br>◯ Don't know
What is the name of the center?	______________________________
List the link to the Center's home page:	______________________________
Does the program require a practicum/applied practice experience? This includes a field placement in a practice-based organization where the student works on activities pertinent to the organization.	◯ Yes<br>◯ No<br>◯ Don't know
Does the program require a capstone/integrated learning experience/culminating experience? This includes working with a practice-based organization to apply analytical skills developed through the student's education.	◯ Yes<br>◯ No<br>◯ Don't know
Does the program require a thesis? This includes work on a specific research question.	◯ Yes<br>◯ No<br>◯ Don't know
Notes about the specific program:	_____________________________________
